# Effects of Accentuated Eccentric and Maximal Strength High-Resistance Training Programs with or Without a Curcumin-Based Formulation Supplement on Body Composition, Blood Pressure, and Metabolic Parameters in Older Adults

**DOI:** 10.3390/diseases13020062

**Published:** 2025-02-18

**Authors:** Alvaro Juesas, Angel Saez-Berlanga, Carlos Babiloni-Lopez, Ezequiel G. Martin, Luis Garrigues-Pelufo, Ana Ferri-Caruana, Javier Gene-Morales, Fernando Martin-Rivera, Iván Chulvi-Medrano, Pablo Jiménez-Martínez, Carlos Alix-Fages, Magdalena Cwiklinska, Veronica Gallo, Virginia Zarza, Pedro Gargallo, Julio Fernandez-Garrido, Oscar Caballero, Jose Casaña, Elisa Moretti, Elisa Grazioli, Giovanni Angelo Navarra, Marianna Bellafiore, Danica Janicijevic, Raouf Hammami, Juan C. Colado

**Affiliations:** 1 Department of Education Sciences, CEU Cardenal Herrera University, 46115 Castellón, Spain; alvaro.juesastorres@uchceu.es; 2 Department of Physical Education and Sports, University of Valencia, 46010 Valencia, Spain; angel.saez@uv.es (A.S.-B.); fernando.martin-rivera@uv.es (F.M.-R.); ivan.chulvi@uv.es (I.C.-M.); juan.colado@uv.es (J.C.C.); 3 Research Group in Prevention and Health in Exercise and Sport (PHES), University of Valencia, 46010 Valencia, Spain; cbabilon@uji.es (C.B.-L.); ezequielgmnutricion@gmail.com (E.G.M.); luisgape@alumni.uv.es (L.G.-P.); p.jimenez@icen.es (P.J.-M.); c.alix@icen.es (C.A.-F.); pedro.gargallo@uv.es (P.G.); 4 ICEN Institute, 28002 Madrid, Spain; calidad@indiex.es (M.C.); vgallo@nutris.es (V.G.); vzarza@nutris.es (V.Z.); 5 Nursing Department, Faculty of Nursing and Podiatry, University of Valencia, 46010 Valencia, Spain; julio.fernandez@uv.es (J.F.-G.); oscar.caballero@uv.es (O.C.); 6 Exercise Intervention for Health Research Group (EXINH-RG), Physiotherapy Department, University of Valencia, 46010 Valencia, Spain; jose.casana@uv.es; 7 Department of Movement, Human and Health Science, Faculty of Sport Science, University of Rome “Foro Italico”, 00135 Rome, Italy; e.moretti1@studenti.uniroma4.it (E.M.); elisa.grazioli@uniroma4.it (E.G.); 8 Sport and Exercise Sciences Research Unit, Department of Psychology, Educational Science and Human Movement, University of Palermo, 90133 Palermo, Italy; giovanniangelo.navarra@gmail.com (G.A.N.); marianna.bellafiore@unipa.it (M.B.); 9 Faculty of Sports Science, Ningbo University, Ningbo 315211, China; jan.danica@gmail.com; 10 Department of Radiology, Ningbo No. 2 Hospital, Ningbo 315010, China; 11 Department of Sports Sciences and Physical Conditioning, Faculty of Education, Universidad Católica de la Santísima Concepción, Concepción 4090541, Chile; 12 Tunisian Research Laboratory ’Sports Performance Optimization’ (CNMSS-LR09SEP01), National Center of Medicine and Science in Sports (CNMSS), Tunis 1004, Tunisia; raouf.cnmss@gmail.com; 13 Higher Institute of Sport and Physical Education of Ksar Said, Manouba University, Manouba 2010, Tunisia

**Keywords:** elastic bands, rating of perceived effort, strength training, fat and muscle mass, glycemia, creatinine, lipid profile

## Abstract

Background/Objectives: This study compared the effects of high-resistance training (RT) programs, with or without curcumin supplementation, on variables commonly associated with metabolic syndrome (MetS), including body composition, blood pressure, and metabolic parameters. Methods: Eighty-one older adults at risk of MetS (BMI > 25 kg/m^2^, fat mass > 32%, and systolic blood pressure > 130 mmHg in half of the participants) were randomly assigned to six groups, which were comprised as follows: four experimental groups involving either accentuated eccentric or maximal strength RT with curcumin or placebo and two control groups receiving either curcumin or placebo. Experimental groups completed a 16-week full-body RT with elastic bands, while controls did not exercise. Results: The results showed that (I) all experimental protocols significantly reduced fat mass (*p* ≤ 0.001), with the maximal strength RT group supplemented with curcumin (Max-Cur) showing the greatest reduction, at 14.3%; (II) muscle gains were significant and comparable across experimental groups (*p* ≤ 0.008); (III) both systolic and diastolic blood pressure decreased similarly across experimental groups (*p* ≤ 0.001); (IV) metabolic parameters significantly improved across experimental groups (*p* ≤ 0.037), except for creatinine, which showed an undesirable peak only in the Max-Cur group; (V) curcumin supplementation enhanced the effects of both RT programs; and (VI) between 54% and 100% of participants achieved clinically meaningful improvements in seven out of ten MetS-related variables across experimental groups. Conclusions: Our findings indicate that high-RT programs combined with curcumin supplementation positively impacted all MetS-related variables. The Max-Cur RT group stood out as the most beneficial, with the greatest fat mass reductions, highlighting this approach as a promising strategy to reduce the risk of MetS in older adults.

## 1. Introduction

Metabolic syndrome (MetS) is a major global health problem characterized by a cluster of clinical, metabolic, and biochemical abnormalities such as obesity, hypertension, insulin resistance, and dyslipidemias [1]. These abnormalities collectively elevate the risk of developing cardiovascular diseases, type 2 diabetes, and renal events, among others [1,2]. The prevalence of MetS is rising worldwide, particularly among older adults [2]. This trend is largely attributed to the natural age-related decline in the function of organs directly involved in the development and progression of MetS, and it is aggravated by a sedentary lifestyle [2,3]. Normal cellular oxidative–metabolic reactions (endogenous) and other factors (exogenous), such as radiation, food constituents, tobacco smoke, and environmental pollutants, form free radicals and other reactive oxygen and nitrogen species (ROS and RNS), and generate MetS [4,5]. Detecting MetS is challenging due to the absence of clear initial symptoms and variability in diagnostic criteria, such as differing BMI thresholds set by the World Health Organization (≥30 kg/m^2^) and American Association of Clinical Endocrinologists (≥25 kg/m^2^) [1]. Adopting preventive strategies is the best ally, as managing MetS becomes significantly more challenging once it develops and causes a serious decline in quality of life [6].

Increasing physical activity levels is thought to be one of the most effective strategies for preventing and managing MetS, as it targets the core of cluster abnormalities associated with the condition, including obesity, hypertension, insulin resistance, and dyslipidemia [7]. This reasoning is empirically supported by numerous studies showing that participating in structured exercise programs can significantly reduce the risk and improve the management of MetS [8,9,10]. In this context, aerobic exercise programs stand out as some of the most commonly implemented strategies [8,10]. In contrast, the potential of alternative exercise approaches, such as resistance training (RT), remains relatively underexplored [11]. This gap is unexpected, given that RT programs have demonstrated significant positive effects on variables associated with all related MetS variables. These include improvements in body composition through increased muscle mass and reduced fat mass [12,13], reductions in resting blood pressure, and enhancements in fasting glucose levels and lipid profiles [12,14,15,16]. Despite the demonstrated benefits of RT programs for addressing MetS-related variables, most studies have focused on low- to moderate-intensity protocols, leaving the potential effects of high-intensity RT largely unexplored [12,13,14,15,16].

Although the impact of high-RT protocols on MetS-related variables in older adults remains largely underexplored, several studies have confirmed their safety and superior effectiveness in enhancing strength compared to low- and moderate-intensity RT programs [17,18,19]. Moreover, greater strength gains have been positively linked to a reduced prevalence of MetS [7]. This raises the question of whether the superior strength improvements achieved through high-intensity RT could also drive meaningful changes in MetS-related variables. Additionally, identifying the most effective high-RT protocol is essential to provide tailored and precise recommendations for older adults in managing MetS. Typical high-RT protocols applied in older adults consist of constant-load gym-based exercises performed using either submaximal or maximal loadings during concentric–eccentric regimens [20]. However, emerging evidence indicates that accentuated eccentric (Aecc) RT may surpass traditional constant-load methods in effectiveness for building strength. The main characteristic of the Aecc protocols is the application of heavier loads during the eccentric compared to the concentric phase [21]. This approach leverages the skeletal muscles’ greater force-generating capacity during eccentric movements, providing a more intense and targeted training stimulus than conventional constant-load protocols [22,23]. Despite its potential, research on Aecc RT has been limited to athletes and healthy young individuals, leaving its application and benefits for older populations unexplored [21]. Investigating the efficacy of Aecc RT in older adults could pave the way for innovative strategies to enhance MetS management in this demographic.

A possible reason why RT is often avoided by researchers is due to its negative perception among older adults, who frequently view it as overly demanding and associated with a high risk of injury [24]. Additionally, its association with gym-based settings can make RT less appealing and more challenging to stick to, especially compared to more accessible aerobic exercises like brisk walking or jogging. However, RT can be effectively adapted to non-gym environments. Among various alternatives, elastic bands offer a practical solution, being more user-friendly, portable, and cost-effective than gym-based equipment [25]. Importantly, numerous studies have demonstrated that RT programs using elastic bands, when matched in intensity, are just as effective as gym-based machine exercises in promoting strength gains [24,26,27,28]. Elastic bands also have the advantage of addressing the “sticking point”, the phase of movement where the exercise becomes most challenging due to biomechanical disadvantage, an issue commonly encountered in constant-load exercises on gym machines [29]. Additionally, elastic band training has been proven to be effective in addressing MetS-related variables, including improving body composition [30], blood pressure [31], and the lipid profile in older adults [30,32]. However, similar to traditional RT programs, most of these findings stem from studies using low-to-moderate RT protocols. As a result, it remains unclear whether high-RT with elastic bands could yield comparable or even greater benefits [28,30,31,32].

To enhance the prevention and management of MetS, many studies recommend combining increased physical activity with targeted supplementation [33]. Various supplements have been developed to address specific abnormalities associated with MetS. For instance, green tea and omega-3 fatty acids have demonstrated effectiveness in lowering resting blood pressure, triglycerides, total cholesterol, and low-density lipoprotein (LDL), while improving high-density lipoprotein (HDL) levels [34,35,36]. Other supplements, such as conjugated linoleic acid [37] or resveratrol [38], have shown greater success in other aspects of MetS, such as reducing fat mass and weight management. While the vast majority of supplements focus on addressing one or two MetS-related aspects, curcumin supplementation stands out as a particularly viable approach, demonstrating beneficial effects across all major factors associated with MetS [11]. These include reductions in fat mass [39], resting blood pressure [40], fasting blood sugar [41], and improvements in lipid profile parameters, such as decreased total cholesterol, triglycerides, and LDL cholesterol, alongside increased HDL cholesterol levels [40]. However, the majority of these findings come from individual studies with diverse participant groups, limiting the generalizability of achieving all of these benefits within a single population.

Physical exercise and curcumin supplementation share certain mechanisms that are positive for the management of METs [42,43,44,45,46]. For instance, both interventions help to regulate the lipidic profile through increased activity of lipoprotein lipase and activation of lipid metabolic pathways, which increases fatty acid oxidation [42,43,47]. Both interventions also help regulate the glycemic profile and insulin resistance by inhibiting liver gluconeogenesis through the modulation of 5′AMP-activated protein kinase and increasing insulin-mediated glucose consumption by activation of glucose transporter type 4 [42,44,45]. Other mechanisms include modulation of vascular aspects that help to prevent cardiovascular disease, atherosclerosis, and endothelial dysfunction through a reduction in reactive oxygen species; an increase in the serum activities of antioxidants, such as superoxide dismutase, and regulation of endothelin 1; modulation of immune responses by inhibition of regulatory T cells and stimulation of effector T cells; and the reduction of inflammatory cytokines and increase of anti-inflammatory cytokines [42,44,46,48,49]. Therefore, it would be valuable to explore whether curcumin supplementation combined with RT protocols can produce a synergistic effect and amplify the positive effects on MetS-related variables in healthy older adults.

To address the identified research gaps, this study aimed (I) to compare the effectiveness of two high-RT protocols (accentuated eccentric [Aecc] and traditional concentric–eccentric [Max]) in improving MetS-related variables (i.e., body composition, resting blood pressure, and metabolic parameters) and (II) to investigate whether combining high-RT elastic band programs with curcumin supplementation can further enhance these improvements. We hypothesized that (I) Aecc RT will produce greater improvements in MetS-related variables compared to Max RT and that (II) combining high-RT programs with curcumin supplementation will yield synergistic effects, leading to more significant improvements in MetS-related variables than high-RT or curcumin supplementation alone. The findings of this study may help to identify the most suitable high-RT regimen for older adults at risk of developing MetS and determine whether its benefits can be further enhanced with curcumin supplementation.

## 2. Materials and Methods

### 2.1. Participants

Participants were recruited by advertisements posted on the web and mailing lists of the Nau Gran (University of Valencia) and Senior University (Polytechnic University of Valencia). Once a participant showed their interest in participating, we used the snowball technique, asking the person to share the recruitment script with relatives or friends who may be interested in participating. An a priori analysis using G*Power (version 3.1.9.3) determined that a sample size of 60 participants was required for a two-way ANOVA with six groups, ensuring 80% statistical power, an effect size of 0.25, and accounting for a 5% type I error and a 20% type II error. Of the 287 volunteers initially interested, 103 were referred to other studies, and 92 were excluded for meeting one or more exclusion criteria or declining to participate. Meanwhile, 92 met the inclusion criteria and were enrolled in the study. Ultimately, 81 participants completed the entire study (age: 68.2 ± 4.6 years; body mass index: 26.4 ± 4.8 kg/m^2^, see Table 1 for details). Participants included in this study were classified as being at risk for MetS, based on a BMI exceeding 25 kg/m^2^ and systolic blood pressure readings above 130 mmHg in three experimental groups, or near this threshold in the other three groups [1]. All dropouts were due to training adherence issues, with no cases attributed to injuries or adverse responses to the exercise or supplementation program. Participants were randomly assigned to an accentuated eccentric training + curcumin supplementation group (Aecc-Cur; *n* = 16), an accentuated eccentric training + placebo group (Aecc-Pla; *n* = 13), a maximum strength training + curcumin supplementation group (Max-Cur; *n* = 10), a maximum strength training + placebo group (Max-Pla; *n* = 10), a control + curcumin supplementation group (C-Cur; *n* = 15), or a control + placebo group (C-Pla; *n* = 17). To ensure researcher blinding, external staff assigned an alphanumeric code to each participant and training group. Although participants were aware of their group assignment due to their active involvement in the exercises, they were unaware of the protocols followed by the other groups or whether they received curcumin supplementation or a placebo. Participants were eligible for inclusion in the study if they met the following criteria: (i) sedentary adults aged 60 or older, (ii) functionally independent (able to walk 100 m unaided and climb 10 steps without resting), (iii) held a medical certificate confirming suitability to enroll in RT, (iv) had refrained from taking antioxidant supplements (e.g., vitamins C, E, A, omega-3, etc.) for at least six weeks before the study began, and (v) were non-smokers and non-alcoholics. Exclusion criteria included the following: (i) any disease or condition that would prevent safe participation in RT or that could interfere with the safe intake of the study supplement, (ii) severe visual or hearing impairments, (iii) a history of malignant neoplasms or a terminal illness with a life expectancy of less than a year, (iv) significant body weight changes (≥10% within the past year), (v) use of medications within the last six months or currently that could influence the study outcomes (e.g., hormone replacement therapy, calcitonin, corticosteroids, glucocorticoids, allopurinol, etc.), (vi) a score below 23 on the Mini-Mental State Examination, or (vii) participation in another study involving dietary, exercise, or pharmaceutical interventions within the last six months. All potential participants were informed of the study’s purpose, procedures, benefits, risks, and potential discomforts before enrollment. Informed consent was obtained from each participant, who retained the right to withdraw from the study at any time.

The study was conducted in accordance with the Declaration of Helsinki, and approved by the Ethics Committee of University of Valencia (protocol code 1861154, 5 May 2022), and was registered at ClinicalTrials.gov (NCT06620666). This study is part of a larger project aimed at identifying the effects of different types of physical exercise and dietary supplements on the physical performance and biological, psychological, and emotional health of older adults.

### 2.2. Study Design

**Figure 1:**
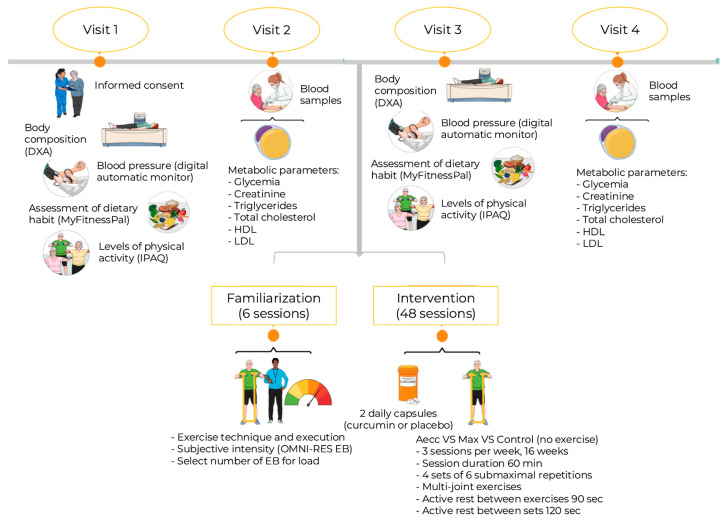
Graphical summary of the study procedures. Created with Mindthegraph.com. Aecc: accentuated eccentric training group. DXA: Dual x-ray absorptiometry. EB: elastic bands. HDL: high-density lipoprotein cholesterol. IPAQ: International Physical Activity Questionnaire. Max: maximum strength training group. LDL: low-density lipoprotein cholesterol. Min: minutes. OMNI-RES EB: OMNI-Resistance Exercise Scale for elastic bands. Sec: seconds.

A longitudinal study was designed to explore the effects of two high-RT programs (Aecc vs. Max) and curcumin supplementation on body composition, blood pressure, and metabolic parameters in older adults. Four experimental groups (Aecc-Cur, Aecc-Pla, Max-Cur, Max-Pla) completed (I) initial testing, (II) six familiarization sessions, (III) a 16-week strength training program, and (IV) a final testing session. During the 16-week intervention, participants completed 48 RT sessions (3 sessions per week), each lasting approximately 60 min. High-RT sessions were conducted on non-consecutive days, with a minimum rest interval of 48 h between them. In contrast, two control groups (C-Cur and C-Pla) only participated in the initial and final testing, without undergoing familiarization or RT training. Both the initial and final testing included body composition analysis, blood pressure measurements, and metabolic parameter assessment through blood sampling. All testing and training sessions were conducted at approximately the same time of day (within a 1 to 2 h window) under consistent environmental conditions. Figure 1 summarizes the study procedures.

### 2.3. Testing Procedures

Initial and final testing sessions were conducted at the University of Valencia by blinded staff uninvolved in the high-RT protocol. Participants were instructed to fast, avoid caffeine for 8 h, and refrain from intensive exercise for 24 h before testing. Initial testing was conducted before the familiarization sessions for the high-RT groups, with final testing carried out within 48 h post-intervention after the completion of the 16-week intervention duration. Firstly, participants were scheduled to attend the Performance Laboratory of the Faculty of Physical Activity and Sports Sciences between 8:30 and 10:00 a.m. Upon arrival, participants rested in a chair for 5 min to standardize baseline conditions. Afterward, their height was measured to the nearest 0.1 cm using a portable stadiometer (Seca GmgH & Co. KG, Hamburg, Germany), while their body weight, fat percentage, fat mass, and muscle mass were assessed using dual-energy X-ray fan beam absorptiometry (QDR^®^ Hologic Discovery Wi, Hologic Inc., Waltham, MA, USA) equipped with APEX software (APEX Corp., version 12.4, Waltham, MA, USA). For these purposes, participants wore light clothing and removed any metals. Blood pressure was then measured with the participants seated in the comfortable chair with both feet resting on the ground and arms resting lightly on a table. The cuff of the digital automatic blood pressure monitor (M2 HEM-7143-E, Omron Healthcare, Kyoto, Japan) was placed on the left arm following the manufacturer’s instruction and the average of two measurements was considered for subsequent analysis.

After completing body composition and blood pressure measurements, participants were taken to the Faculty of Nursing, where qualified nurses collected their blood samples. Blood samples were collected through two vacutainer SSTs (i.e., serum-separated tubes that contain a gel at the bottom to separate blood cells from serum during centrifugation). The samples were centrifuged at 1500 rpm for 10 min at room temperature, while the supernatants were aliquoted and stored at −60 °C until analysis. The photometric ARCHITECT c16000 analyzer (Abbott Diagnostics, IL, USA) was used for analyzing glucose, cholesterol, triglycerides, HDL, and creatinine levels, while LDL levels were estimated using the Friedewald formula as modified by Delong et al. [50]. Procedures followed the Clinical and Laboratory Standards Institute (CLSI) guidelines. Following initial testing, participants in the experimental groups completed six familiarization sessions designed to (i) master proper exercise techniques, (ii) practice subjective intensity evaluation using the OMNI-RES scale for elastic bands [51], (iii) become accustomed to the resistance levels of differently colored elastic bands, and (iv) learn the appropriate velocity and repetition frequency for exercise execution.

### 2.4. Training Procedures

Both high-RT programs included a total of 48 sessions, conducted three times per week over 16 weeks. Each training session lasted approximately 60 min and followed the American College of Sports Medicine guidelines, including the following three components: a 5–10-min general warm-up, a 40–50-min main workout, and a 5–10-min cool-down focusing on flexibility [52]. The main phase of the training involved performing 4 sets of 6 submaximal repetitions of multi-joint exercises. The exercises included lunges, standing horizontal chest presses, standing horizontal hip hinges, and standing horizontal rows, performed in that order. These movements were specifically chosen to engage both major and minor muscle groups and were consistently performed using CLX elastic bands (TheraBand^®^; Hygenic Corporation, Akron, OH, USA) [52]. The rest period between exercises was 90 s, while the interval between sets of the same exercise was 120 s. To enhance participant motivation and ensure adherence to the training program, rest periods were made active, incorporating low-intensity rhythmic coordination exercises and simple cognitive tasks, such as dance movements, reaction time drills, or responses to pre-learned stimuli.

During the exercise execution, participants attached the center of an elastic band to a wall-mounted support and held the ends. The intensity (i.e., resistance used) for the four multi-joint exercises was adjusted both objectively and subjectively. Objectively, resistance was modified using bands with different tension levels, either by employing a single band or combining multiple bands, and/or also by altering the distance from the wall, as follows: closer reduced tension, while farther increased it. Subjectively, the intensity was adjusted by having participants rate the perceived exertion of the first repetition of the set using the OMNI-RES scale for elastic bands. Once optimal intensity (i.e., resistance used) was determined in the first set, an individualized marker was positioned on the floor to allow for maintaining the same exercise intensity across the remaining sets. If adjustments were needed, the distance to the wall and the type or number of elastic bands were modified, and the floor marker was also repositioned according to the new requirements. In accordance with the manufacturer’s safety guidelines, elastic bands were stretched to a maximum of 300% of their resting length to prevent breakage. Upon reaching this limit, the band was replaced with one of greater viscoelastic stiffness to ensure higher resistance. A metronome was used to control the repetition frequency [51]. A qualified sports scientist supervised sessions to ensure proper technique was utilized. Both Aecc and Max RT programs followed guidelines for older adults [53], while the specificities of technique execution of each program are detailed below.

#### 2.4.1. Accentuated Eccentric Elastic Band Program (Aecc)

While being close to the wall, participants performed the concentric phase of the corresponding exercise while the elastic band was not tensed (e.g., performing arm flexion at shoulder-width during the standing horizontal chest presses exercise while holding the elastic band). Afterward, participants started moving farther from the wall until they were unable to maintain the stability or previously undertaken joint position (e.g., unwanted elbow flexion). When this extreme position was reached, the reference marker was positioned on the floor. Then, the participants would start the eccentric phase of the movement (e.g., controlled elbow flexion), which lasted 5 s. This setup ensured that participants trained at 100% of their maximal capacity (i.e., the maximal resistance possible for a concentric phase) while reporting a perceived effort of 7 or 8 out of 10 immediately after completing each of the required eccentric actions throughout the entire series. When they completed the eccentric phase of the movement, they were instructed to return close to the wall and, without pausing, move forward again to the starting position within the 2 s window, then proceed to perform another repetition in the same manner.

#### 2.4.2. Maximal Strength Elastic Band Program (Max)

Participants initially positioned themselves near the wall anchor and gradually moved away to generate sufficient tension in the elastic band, ensuring that the target Rating of Perceived Exertion (RPE) was felt in the active muscles during the first repetition, which was set at an RPE of 8 for all participants [51]. For those who experienced difficulties in perceiving active muscle effort, an RPE of 7 for the overall body was used [25]. Both RPEs of 7 and 8 corresponded to the intensity of approximately 85% of their one-repetition maximum (1RM) [51]. Once participants achieved the desired tension, they performed both the concentric (e.g., elbow extension) and eccentric (e.g., elbow flexion) phases of the exercise. If the perceived difficulty of the movement did not match the target RPE, participants adjusted their distance from the wall to either increase or decrease elastic band tension. After finding the optimal position, a floor marker was placed to ensure consistency in subsequent repetitions. The duration of both the concentric and eccentric phases was standardized to 2 s each, and participants did not reach an RPE of 10 by the end of the set.

### 2.5. Supplementation Procedures

Participants were randomly assigned to receive either two daily capsules of curcumin or a placebo. Starting from the first day of intervention, one capsule was taken at 8:00 am after breakfast and the other at 9:30 pm after dinner. Each curcumin capsule contained 250 mg of Curcumin extract CursolTM (Nutris Ingredients, Madrid, Spain), standardized to 2.1% (5.25 mg) curcumin, with the dosage aligned to established recommendations for human consumption [54,55,56]. CursolTM is composed of dibasic calcium phosphate anhydrous (E341-stabilizer), Polysorbate 80 (E433-emulsifier), turmeric rhizome extract (*Curcuma longa* L.), and citric acid (E330-acidity regulator). Therefore, the daily total dose of the study was 500 mg of the product or the placebo. Placebo capsules, identical in appearance, contained maltodextrin and excipients. Both participants and investigators remained blinded to supplementation conditions throughout the study.

### 2.6. Dietary and Physical Activity Control

Participants were instructed to maintain their usual dietary habits throughout the study to avoid influencing the outcome measures. To ensure compliance, dietary habits were assessed at the start and end of the training program using a self-reported diet record covering two weekdays and one weekend day, recorded via the validated smartphone app MyFitnessPal (MyFitnessPal, LLC, CA, USA) [57,58]. A nutritionist experienced in RT guided participants on the correct use of the app and monitored their dietary intake throughout the study. The physical activity levels of each participant were measured pre- and post-intervention through the self-reported International Physical Activity Questionnaire (IPAQ). We ensured that participants evaluated their physical activity levels without considering their participation in the exercise sessions of the study.

### 2.7. Statistical Analysis

Descriptive data are presented using mean and standard deviations (SD). The Shapiro–Wilk test revealed that the creatinine, triglycerides, and total cholesterol were the only variables that deviated from normal distribution. Percent difference (Δ% = [post-test score–pre-test score]/pre-test score] × 100) was used to quantify the differences in the magnitude of the dependent variables between the pre-test and post-test. A two-way repeated measures analysis of variance (ANOVA) was conducted with time as a within-subject factor (pre-test vs. post-test) and group as a between-subject factor (Aecc-Cur vs. Aecc-Pla vs. Max-Cur vs. Max-Pla vs. C-Cur vs. C-Pla) on the normally distributed variables, which were equalized between groups at the baseline (i.e., muscle mass, systolic and diastolic blood pressure). Subsequently, a two-way analysis of covariance (ANCOVA) was employed on normally distributed variables that were not equalized between the groups at the beginning of the study. This was completed using baseline values of fat mass, glycemia, HDL, and LDL as covariates to eliminate the potential influence of initial differences on intervention outcomes [59,60]. In cases where a significant F-ratio was obtained, 95% confidence intervals were applied to examine the differences between the adjusted ANCOVA pre- and post-test scores within groups, as well as the post-test differences between groups. Finally, the Least Significant Difference (LSD) test was employed for post hoc comparisons, with effect sizes (ES) evaluated using partial eta squared (ƞp^2^).

The Kruskal–Wallis test revealed significant differences in both the pre-test and post-test for all variables that deviated from normal distribution (creatinine, triglycerides, and total cholesterol). The magnitude of these differences was assessed using ƞp². Then, Quade’s rank analysis of covariance (Quade’s ANCOVA) was applied using the baseline values of these variables as covariates to adjust for pre-existing differences enhancing the precision of comparisons across groups [61,62]. When the test statistic reached statistical significance, 95% confidence intervals were used to determine the presence of meaningful differences between the adjusted Quade’s ANCOVA means pre- and post-intervention stages, as well as post-intervention differences between groups. The Friedman Test assessed the effects of time as the within-participant factor, with ES evaluated using Kendall’s Coefficient of Concordance (*w*) following Cohen’s guidelines [63]. Post hoc comparisons were conducted using Wilcoxon tests for within-group analyses and Mann–Whitney U-tests for between-group analyses, with ES calculated using Hedge’s g.

The number of participants who reached the minimal clinically important difference (MCID) end-point for each dependent variable, was calculated manually. The MCID is defined as (i) the minimally important difference or change, (ii) as well as the minimally detectable difference or change in the variable of interest that patients perceive as beneficial, warranting a treatment modification in the absence of significant side effects or excessive cost [64,65]. It is important to note that researchers are advised against using previously established MCID cut-off values in new trials unless the intervention, outcome measure, and patient population closely align with the original settings in which the MCID was defined [66]. Missing data in this study were addressed using multiple imputations, specifically following the intention-to-treat method, where baseline measurements for participants who withdrew were carried forward to the post-intervention phase [67]. The qualitative interpretation of ƞp^2^ was as follows: values between 0.01 and 0.06 indicated a small effect, values from 0.06 to 0.14 represented a medium effect, and values greater than 0.14 corresponded to a large effect. ES was calculated using Hedge’s *g* to account for a potential small sample bias and was interpreted based on Cohen’s guidelines, as follows: trivial effect (<0.20), small effect (0.20–0.50), moderate effect (0.50–0.80), and large effect (>0.80) [63]. Statistical analyses were performed using the software package SPSS (IBM SPSS version 28.0.1.1(14), Armonk, NY, USA). Alpha was set at a *p* < 0.05 level.

## 3. Results

**Figure 2:**
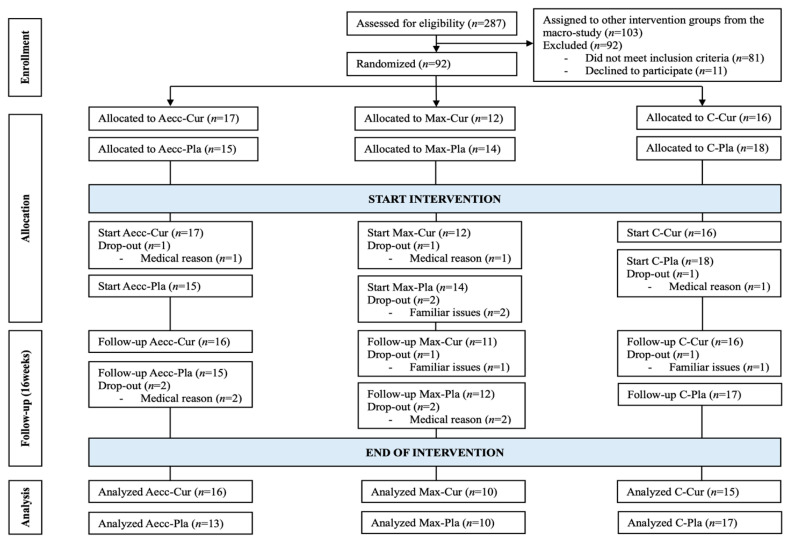
Participant flow throughout the study.

The participant flow through the study can be seen in Figure 2. Of the initial 287 participants that responded to the call, 81 were excluded due to not complying with selection criteria and 14 declined to participate after undergoing an explanation of the specific procedures. After these 95 exclusions, 103 participants were assigned to other intervention groups contained in the macro-study and 92 started the specific procedures presented in this manuscript. Out of these 92 participants, 81 completed the whole study. Participants’ characteristics are presented in Table 1. No significant differences were found between groups in terms of age, weight, height, BMI, body fat percentage, weekly physical activity, or dietary intake (total calories, protein, carbohydrates, and lipids) at baseline or post-intervention (*p* > 0.161 for all variables). Similarly, no significant changes in dietary intake (total calories, protein, carbohydrates, and lipids) and physical activity levels were observed from pre- to post-intervention in any of the groups (all *p* > 0.152). The average attendance rate at the end of the training program was comparable across all groups, as follows: 89.58% (43 of 48 sessions) for Aecc-Cur, 87.50% (42 of 48 sessions) for Aecc-Pla, 87.23% (41 of 47 sessions) for Max-Cur, and 89.36% (42 of 47 sessions) for Max-Pla. Reported reasons for missed sessions included family obligations, medical appointments, spouse’s health, and transportation issues; none of the participants reported injuries or adverse events derived from the study procedures. Self-reported adherence to the supplementation protocol was 89.39%.

### 3.1. Body Composition

The interaction time × group was significant both for fat mass (F = 65.13, *p* < 0.001, ηp^2^ = 0.82) and for muscle mass (F = 37.47, *p* < 0.001, ηp^2^ = 0.72), showing greater fat mass loss and higher muscle gains in experimental groups compared to the control groups. A significant effect of time was found for the fat mass (F = 4.67, *p* = 0.030, ηp^2^ = 0.06) and muscle mass (F = 10.06, *p* = 0.002, ηp^2^ = 0.12). All of the training groups significantly reduced their body fat and increased their muscle mass, showing significant differences to the controls. The control groups significantly increased their body fat and decreased their muscle mass. Pairwise comparisons revealed the highest fat loss in the Max-Cur group, while no differences in the muscle mass gains were obtained between experimental groups (Table 2).

### 3.2. Blood Pressure

The interaction time × group was significant for both systolic blood pressure (F = 23.93, *p* < 0.001, ηp^2^ = 0.62) and diastolic blood pressure (F = 18.92, *p* < 0.001, ηp^2^ = 0.56), revealing differing trends between the experimental and control groups, with systolic and diastolic blood pressure decreasing after the training program in the experimental groups but slightly increasing in the control groups. The main effect of time was significant for both the systolic blood pressure (F = 160.47, *p* < 0.001, ηp^2^ = 0.68) and diastolic blood pressure (F = 85.46, *p* < 0.001, ηp^2^ = 0.54). Pairwise comparisons were presented in Table 3. The most noteworthy results were that all of the training groups significantly reduced their systolic and diastolic blood pressure, and that all of the groups had significantly lower systolic blood pressure compared to the controls, but only participants in the Max-Cur group had significantly lower diastolic blood pressure compared to the controls. While the controls taking a placebo significantly increased their systolic and diastolic blood pressure, the controls taking curcumin did not have significant changes.

### 3.3. Metabolic Parameters

The interaction time × group was significant for all metabolic parameters (glycemia: F = 9.24, *p* < 0.001, ηp^2^ = 0.39; triglycerides: H = 13.53, *p* = 0.0019, ηp^2^ = 0.17; total cholesterol: H = 20.82, *p* < 0.001, ηp^2^ = 0.27; HDL: F = 13.17, *p* < 0.001, ηp^2^ = 0.48; and LDL: F = 9.31, *p* < 0.001, ηp^2^ = 0.39), except for creatinine (H = 8.04, *p* = 0.154). The main effect of time was significant for glycemia (F = 7.67, *p* = 0.007, ηp^2^ = 0.10), triglycerides (χ^2^ = 16.61, *p* < 0.001, W = 0.21), total cholesterol (χ^2^ = 29.54, *p* < 0.001, W = 0.37), and HDL (F = 9.00, *p* = 0.004, ηp^2^ = 0.58), while it was not significant for creatinine (χ^2^ = 0.65, *p* = 0.419) and LDL (F = 0.72, *p* = 0.400). The pairwise comparisons are presented in Table 4. All of the training groups significantly reduced their glycemia, triglycerides, and total cholesterol, showing significant differences to the controls. It is to be noted that Aecc-Cur and Max-Cur significantly increased their creatinine levels, while the Max-Cur group showed significant differences to both the Aecc groups and the controls.

### 3.4. Clinical Relevance

At least half of the participants in the four experimental groups (Aecc or Max combined with supplement or placebo) achieved end-points in at least 7 out of the 10 dependent variables, indicating benefits in reducing cardiovascular risk and mortality (Table 5).

## 4. Discussion

The present study aimed to identify the most effective high-intensity RT strategy for improving body composition, lowering resting blood pressure, and globally enhancing metabolic parameters, which are key factors linked to an elevated risk of developing MetS. The main findings of this study revealed that (I) all high-RT protocols resulted in significant reductions in body fat mass and increases in muscle mass following the intervention, while between-group analysis showed that the Max-Cur group achieved superior fat mass reductions compared to other high-RT groups, while muscle mass gains were comparable across all groups and ranged from 1.4% to 2.3%. (II) Significant and comparable reductions in systolic blood pressure (ranging from 9.8% to 11.4%) and diastolic blood pressure (ranging from 6.3% to 10.4%) occurred across all high-RT groups. (III) All metabolic parameters improved significantly following the high-RT programs, with similar improvements observed across the experimental groups. The only exception to this trend was creatinine levels, which were significantly elevated post-test in the Max-Cur group compared to the other experimental groups. (VI) Curcumin supplementation accentuated the positive effects of both high-RT programs for all variables except for creatinine. (V) Finally, importantly, between 54% and 100% participants reached clinically relevant improvements in 7 out of 10 dependent variables across all of the experimental groups. These findings suggest that all high-RT programs provided significant benefits for improving body composition, metabolic parameters, and blood pressure, with the Max-Cur group showing the greatest benefits for fat reduction. Therefore, although both high-intensity RT programs combined with curcumin supplementation achieved similar improvements, the Max-Cur strategy is recommended due to its greater effectiveness in reducing fat mass.

This discussion is developed, first, by evaluating the influence of the interventions on each dependent variable (i.e., body composition, blood pressure, and metabolic parameters) and second, by discussing the potential synergistic effect that curcumin supplementation may have with high-resistance resistance training with elastic bands.

### 4.1. Body Composition

Participants across all experimental groups experienced significant and positive changes in body composition following the intervention, with fat mass reductions ranging from 8% to 14.4% and muscle mass gains between 1.4% and 2.3%. Although the positive effects of high-resistance RT on muscle mass gains and their underlying mechanisms are well-documented and expected [78], its impact on fat reduction has been less extensively explored. A plausible explanation for the effectiveness of high-RT in reducing fat mass, even among older adults at risk of MetS, could be explained by increased energy expenditure during exercise and the temporary elevation in resting metabolic rate following the activity [79,80]. Between-group analysis showed that muscle mass gains were comparable across all experimental groups, whereas the Max-Cur protocol proved to be the most effective strategy for reducing fat mass. The combination of a Max RT protocol, integrating eccentric and concentric contractions at the same high submaximal intensity, together with curcumin supplementation, appeared to enhance energy expenditure more effectively than any other protocol. This could be due to, first, the increased time under tension of performing both phases of the exercise (concentric and eccentric) compared to only the eccentric phase, as a longer time under tension produces greater energy expenditure [81]. Additionally, curcumin supplementation shows increased fatty acid oxidation, resting metabolic rate, reduction of inflammatory cytokines, and regulation of the glycemic profile and insulin resistance in rats and older adults [11]. The combination of these factors may have contributed to maximizing the fat reduction in Max-Cur.

Most importantly, all participants in the Max-Cur group achieved clinically significant thresholds for health benefits, including a minimum 5% reduction in fat mass [68] and muscle mass gains exceeding 1% [82]. These outcomes were not consistently observed with other experimental groups, highlighting the potential of the Max-Cur high-RT regimen as the most beneficial one for improving body composition parameters in older adults at risk of developing MetS.

### 4.2. Blood Pressure

Regarding resting blood pressure, participants in all experimental groups experienced comparable and significant improvements, with systolic reductions ranging from 9.8% to 11.4% and diastolic reductions ranging from 6.3% to 10.4%. Previous meta-analyses have generally reported a positive hypotensive effect of RT [83,84,85], but the reductions observed in our study were of a greater magnitude. For instance, Kelley and Kelley [83] reported decrements of ≈2% for systolic and ≈4% for diastolic blood pressure, while Cornelissen and Fagard [84] reported an absolute decrement of 3.2 mmHg and 3.5 mmHg for the systolic and diastolic blood pressure, respectively. The smaller blood pressure reductions reported in previous meta-analyses compared to our study may be attributed to the inclusion of hypertensive participants, who are less responsive to the hypotensive effects of RT, and the use of isometric RT protocols, which are generally less effective for the blood pressure control [86]. Additionally, most studies included in these meta-analyses involved more heterogeneous age groups, with participants aged 18 years and older, and predominantly utilized moderate-RT protocols, which are considered the most effective for managing blood pressure [85]. However, our findings demonstrate that high-RT programs can also produce significant and positive effects on resting blood pressure without inducing adverse cardiac events in older individuals. Proposed mechanisms for blood pressure reduction with RT include, among others, greater release of nitric oxide and lower adrenergic discharge, resulting in peripheral vascular resistance reduction [48]. The positive effects of curcumin supplementation on blood pressure could be due to improved vascular tone and reduced inflammation, which control endothelial dysfunction [49].

It is worth noting that the exercises in our high-resistance RT program were designed to engage whole-body muscle groups and were executed at a metronome-controlled pace for consistency and safety. Furthermore, our participants were healthy and normotensive, and the use of high resistance was deliberately chosen to elicit broader benefits on all MetS-related variables. The effectiveness of our high-RT programs is further underscored by clinically significant reductions of at least 10% in systolic blood pressure and 5% in diastolic blood pressure, achieved by the vast majority of participants [70].

### 4.3. Metabolic Parameters

All high-RT programs significantly improved resting glycemia and lipid-related parameters, including triglycerides, total cholesterol, HDL, and LDL, with effect sizes ranging from moderate to large. These findings are encouraging, as previous research on the metabolic benefits of RT in older adults has yielded mixed results. For example, some studies reported positive effects of RT programs on lipid parameters after just 8–10 weeks of RT [13,87], whereas others observed no improvements after 12 weeks [16] or even 8 months of RT [88]. The superior findings in our study might be attributed to the implementation of high resistance in our RT programs, distinguishing it from the moderate ones employed in previously cited studies. Another explanation for the positive findings is the favorable changes in body composition observed in our participants, which have been closely linked to improvements in lipid profiles [13]. While all high-RT programs similarly improved glycemia and lipid profiles, the creatinine behavior patterns showed slight variations between groups. Specifically, participants belonging to the Max-Cur group exhibited significantly higher serum creatinine levels post-test compared to all other groups. In contrast, participants in the C-Cur and Max-Pla groups did not experience this peak, suggesting that an unknown mechanism, triggered by the combination of Max high-RT and curcumin supplementation, was responsible for provoking the occurrence of creatinine peak. Although transient elevations in creatinine are a normal response to RT [89], the Max-Cur high-RT protocol should be avoided in individuals with renal dysfunction until it is determined whether this peak may cause significant difficulties in filtering and excreting excess creatinine in this population [90]. Finally, the clinical impact of all high-RT protocols on certain lipid parameters (e.g., triglycerides, total cholesterol, and LDL) was less pronounced compared to their effects on body composition and blood pressure, with clinically significant changes observed in only 0% to 60% of participants across the programs. Extending the duration of high-resistance RT may be necessary to achieve broader and more clinically significant improvements in lipid profiles.

Previous studies suggest that mechanisms for an improved lipidic profile include the increased activity of lipoprotein lipase, suppression of CCAAT/enhancer-binding protein alpha and PPAR expression, and a decrease in cholesterol levels, as well as suppression of the expression of Niemen–Pick C1-like protein in the intestine, which mediates the cholesterol absorption of hepatocytes, among others [42,43,44,47]. Regarding the improvement in the glycemic profile, numerous mechanisms have been proposed, including the inhibition of liver gluconeogenesis through the modulation of 5′AMP-activated protein kinase, which reduces blood glucose levels; as well as the stimulation of insulin-mediated glucose uptake through the phosphatidylinositol 3-kinase/Akt pathway, which, in turn, upregulates glucose transporter type 4 in adipocytes and skeletal muscle [42,44,45].

### 4.4. Synergistic Effects of Curcumin and RT

Supporting our second hypothesis, curcumin supplementation enhanced the effects of both Aecc and Max high-RT protocols, resulting in significantly greater improvements in body composition, blood pressure reduction, and metabolic parameters in groups combining high-RT with curcumin, compared to high-RT alone. A similar trend was observed in the control groups, where curcumin supplementation was more effective in maintaining favorable levels of body composition, blood pressure, and metabolic parameters in the C-Cur RT group compared to the C-Pla RT group. The between-group differences between the two control groups reached statistical significance for LDL levels (*p* = 0.047). The most significant finding is that all previously reported positive effects on individual MetS-related variables—such as reductions in fat mass [39], resting blood pressure [40], fasting blood sugar [41], and improvements in lipid profile [40]—observed in separate studies with different populations were achieved collectively within a single group of older adults at risk of developing MetS. The sole exception to the positive effects of curcumin supplementation was creatinine, which exhibited an undesirable peak when combined with the Max high-RT protocol. As a result, individuals with renal dysfunction should exercise caution when combining curcumin supplementation with the Max high-RT protocol. In summary, our findings coincide with previous research reporting that the synergistic effect of exercise and supplementation with curcumin is caused by different interactions of curcuminoids with other molecules (glucose transporter type 4, endothelin 1, regulatory and effector T cells) that act on different physiological mechanisms and produce, e.g., fat metabolic pathways activation, increased glucose transporters, reduction of oxidative stress and inflammatory cytokines, and immune system modulation [11].

### 4.5. Limitations

Although this study was meticulously conducted using a longitudinal randomized controlled design and it provides highly valuable findings for older adults at risk of MetS, a few limitations should be acknowledged. Firstly, as the sample consisted of older adults with low-risk MetS, the generalizability of the findings to hypertensive or clinically diverse populations is limited. Secondly, the 16-week intervention produced clinically relevant changes in body composition and resting blood pressure for most participants, but fewer participants achieved significant improvements in lipid profiles. Extending the duration of high-RT programs in future studies (for example, session duration and/or the number of weekly sessions) may help to determine whether similar interventions could yield clinically significant improvements in a larger proportion of participants. Finally, the impact of curcumin supplementation on creatinine levels in the Max RT group requires further investigation to determine whether it may have objectively negative effects on individuals with pre-existing renal conditions, or if this effect was a consequence of the interaction with the maximum exercise protocol, as creatinine was not increased when curcumin was ingested in the control group.

## 5. Conclusions

All high-RT programs in this study produced significant improvements in body composition, blood pressure, and metabolic parameters in older adults at risk of MetS. Collectively, our findings indicate that a combination of high-RT programs and curcumin supplementation positively impacted all MetS-related variables without causing adverse health effects, highlighting its potential as a promising strategy for reducing MetS risk in older adults. The Max-Cur group demonstrated superiority in certain aspects, such as greater fat reductions, compared to the other groups. However, creatinine peaks were observed after the Max-Cur RT program and, therefore, caution should be applied in recommending such exercise to individuals with pre-existing renal conditions until the clinical significance of this effect is clarified.

## Figures and Tables

**Table 1:** Baseline characteristics of the participants belonging to the different intervention groups.

Variable	Aecc-Cur (*n* = 16)	Aecc-Pla (*n* = 13)	Max-Cur (*n* = 10)	Max-Pla (*n* = 10)	C-Cur (*n* = 15)	C-Pla (*n* = 17)
Age (years)	68.1 ± 5.1	69.5 ± 4.0	67.0 ± 4.2	68.0 ± 5.2	69.5 ± 4.7	66.9 ± 4.4
Weight (kg)	73.1 ± 11.0	69.5 ± 12.0	73.2 ± 10.7	71.1 ± 11.1	70.7 ± 11.3	70.1 ± 11.7
Height (cm)	167.5 ± 6.7	163.2 ± 7.2	163.0 ± 7.7	165.9 ± 7.0	166.9 ± 4.7	162.7 ± 7.2
Body mass index (kg/m^2^)	26.2 ± 3.1	26.8 ± 2.9	26.3 ± 3.4	25.7 ± 3.4	25.3 ± 4.0	25.8 ± 3.4
Body fat percentage (%)	32.5 ± 6.3	34.4 ± 5.6	36.1 ± 4.9	33.8 ± 5.8	35.7 ± 5.1	33.4 ± 5.4
Weekly physical activity (MET)	830.0 ± 8.5	728.5 ± 10.0	781.2 ± 9.1	804.4 ± 8.8	709.2 ± 10.4	684.0 ± 12.5

Data are presented as mean ± standard deviation. Aecc-Cur, accentuated eccentric training + curcumin supplementation group; Aecc-Pla, accentuated eccentric training + placebo group; Max-Cur, maximum strength training + curcumin supplementation group; Max-Pla, maximum strength training + placebo group; C-Pla, passive control + placebo group; C-Cur, passive control + curcumin supplementation group.

**Table 2:** Intervention effects on the body composition of each group.

	Group	Mean ± SD (Pre-Test)	Mean ± SD (Post-Test)	Δ%	*p*-Value (Time)	ES (Time)	Comparison (Group)	*p*-Value (Group)	ES (Group)
Body fat mass (kg)	(1) Aecc-Cur	23.2 ± 5.9	20.6 ± 6.0	−11.4	**<0.001**	0.42	1–2	0.015	0.09
							1–3	<0.001	0.67
	(2) Aecc-Pla	22.8 ± 4.8	21.0 ± 4.0	−8.0	**<0.001**	0.40	1–5	<0.001	0.51
							1–6	<0.001	0.54
	(3) Max-Cur	28.7 ± 5.7	24.6 ± 5.7	−14.3	**<0.001**	0.69	2–3	<0.001	0.72
							2–4	0.015	0.58
	(4) Max-Pla	27.8 ± 8.5	24.7 ± 8.2	−11.1	**<0.001**	0.35	2–5	0.004	0.48
							2–6	<0.001	0.53
	(5) C-Pla	23.7 ± 7.3	24.3 ± 8.1	2.4	**<0.001**	0.05	3–4	0.013	0.03
							3–5	<0.001	0.04
	(6) C-Cur	23.3 ± 6.5	24.0 ± 6.4	3.1	**0.003**	0.11	3–6	<0.001	0.11
							4–5	<0.001	0.06
							4–6	<0.001	0.12
Body muscle mass (kg)	(1) Aecc-Cur	44.1 ± 8.1	44.9 ± 7.9	1.8	**<0.001**	0.10	1–5	<0.001	0.16
							1–6	0.021	0.11
	(2) Aecc-Pla	43.1 ± 9.9	43.8 ± 10.1	1.4	**0.001**	0.07	2–5	<0.001	0.11
							2–6	<0.001	0.09
	(3) Max-Cur	43.6 ± 8.1	44.6 ± 8.3	2.3	**0.001**	0.12	3–5	<0.001	0.18
							3–6	0.001	0.12
	(4) Max-Pla	39.5 ± 7.3	40.2 ± 7.3	1.7	**0.005**	0.10	4–5	0.001	0.14
							4–6	0.038	0.10
	(5) C-Pla	38.0 ± 6.7	37.8 ± 6.7	−0.7	**0.005**	0.03			
		
	(6) C-Cur	41.6 ± 13.1	41.3 ± 12.9	−0.8	**0.001**	0.03			
		

Data are presented as means ± standard deviations. Significant pairwise time differences are highlighted in bold. Δ% represents the percent change between pre- and post-intervention, calculated as [(post-test score − pre-test score)/pre-test score] × 100. ES, Hedge’s g effect size. Aecc-Cur, accentuated eccentric training + curcumin supplementation group; Aecc-Pla, accentuated eccentric training + placebo group; Max-Cur, maximum strength training + curcumin supplementation group; Max-Pla, maximum strength training + placebo group; C-Pla, passive control + placebo group; C-Cur, passive control + curcumin supplementation group.

**Table 3:** Intervention effects on the blood pressure parameters of each group.

	Group	Mean ± SD Pre-Test	Mean ± SD Post-Test	Δ%	*p*-Value (Time)	ES (Time)	Comparison (Group)	*p*-Value (Group)	ES (Group)
Systolic blood pressure (mmHg)	(1) Aecc-Cur	133.5 ± 17.0	118.2 ± 17.4	−11.4	**<0.001**	0.93	1–5	0.017	0.99
							1–6	0.029	0.76
	(2) Aecc-Pla	130.5 ± 11.8	117.7 ± 12.0	−9.8	**<0.001**	1.07	2–5	0.017	1.08
							2–6	0.032	0.93
	(3) Max-Cur	135.3 ± 13.1	120.2 ± 13.6	−11.2	**<0.001**	1.17	3–5	0.024	0.82
							3–6	0.041	0.69
	(4) Max-Pla	130.5 ± 11.6	117.7 ± 10.4	−9.9	**<0.001**	1.17	4–5	0.018	1.14
							4–6	0.031	0.96
	(5) C-Pla	126.5 ± 13.7	130.2 ± 11.4	2.9	**<0.001**	0.29			
		
	(6) C-Cur	128.3 ± 13.5	129.3 ± 12.9	0.7	0.461	0.08			
		
Diastolic blood pressure (mmHg)	(1) Aecc-Cur	82.3 ± 8.6	76.1 ± 7.9	−7.6	**<0.001**	0.75	3–5	0.018	1.09
							3–6	0.036	0.81
	(2) Aecc-Pla	80.3 ± 7.1	75.2 ± 7.5	−6.3	**<0.001**	0.71			
		
	(3) Max-Cur	83.6 ± 7.5	74.9 ± 7.8	−10.4	**<0.001**	1.10			
		
	(4) Max-Pla	80.2 ± 10.2	73.3 ± 7.4	−8.6	**<0.001**	0.84			
		
	(5) C-Pla	79.0 ± 7.6	80.0 ± 6.3	1.3	**0.005**	0.13			
		
	(6) C-Cur	77.3 ± 5.3	79.3 ± 4.7	2.7	0.061	0.42			
		

Data are presented as means ± standard deviations. Significant pairwise time differences are highlighted in bold. Δ% represents the percent change between pre- and post-intervention, calculated as [(post-test score − pre-test score)/pre-test score] × 100. ES, Hedge’s g effect size. Aecc-Cur, accentuated eccentric training + curcumin supplementation group; Aecc-Pla, accentuated eccentric training + placebo group; Max-Cur, maximum strength training + curcumin supplementation group; Max-Pla, maximum strength training + placebo group; C-Pla, passive control + placebo group; C-Cur, passive control + curcumin supplementation group.

**Table 4:** Intervention effects on the metabolic parameters of each group.

	Group	Mean ± SD (Pre)	Mean ± SD (Post)	Δ%	*p*-Value (Time)	ES (Time)	Comparison (Group)	*p*-Value (Group)	ES (Group)
Glycemia (mg/dL)	(1) Aecc-Cur	90.0 ± 9.9	79.9 ± 7.8	−11.2	**<0.001**	1.13	1–5	<0.001	1.24
							1–6	<0.001	1.28
	(2) Aecc-Pla	87.8 ± 4.7	79.3 ± 5.4	−9.6	**<0.001**	1.67	2–5	<0.001	1.61
							2–6	0.001	1.45
	(3) Max-Cur	91.6 ± 8.7	80.2 ± 10.2	−12.4	**<0.001**	1.20	3–5	<0.001	1.05
							3–6	<0.001	1.10
	(4) Max-Pla	85.1 ± 8.8	79.1 ± 6.9	−7.1	**0.001**	0.76	4–5	0.001	1.49
							4–6	0.014	1.36
	(5) C-Pla	85.5 ± 5.5	88.4 ± 5.6	3.4	0.270	0.53			
		
	(6) C-Cur	93.3 ± 11.8	91.8 ± 10.2	−1.6	0.922	0.14			
		
Creatinine (md/dL)	(1) Aecc-Cur	0.91 ± 0.16	0.93 ± 0.17	2.2	**0.015**	0.12	1–3	0.002	0.97
							1–5	0.023	0.39
	(2) Aecc-Pla	0.92 ± 0.17	0.92 ± 0.16	0.0	0.645	0.00	2–3	0.001	0.93
							3–4	0.027	0.34
	(3) Max-Cur	0.80 ± 0.16	0.94 ± 0.17	17.5	**0.028**	0.85	3–5	<0.001	1.02
							3–6	<0.001	0.44
	(4) Max-Pla	0.83 ± 0.18	0.88 ± 0.17	6.0	0.066	0.29	4–5	0.006	0.68
		
	(5) C-Pla	0.81 ± 0.17	0.76 ± 0.18	−6.2	**<0.001**	0.28			
		
	(6) C-Cur	0.85 ± 0.19	0.86 ± 0.19	1.2	0.207	0.06			
		
Triglycerides (mg/dL)	(1) Aecc-Cur	99.7 ± 36.5	82.62 ± 28.7	−17.1	**<0.001**	0.52	1–5	<0.001	0.28
							1–6	0.002	0.65
	(2) Aecc-Pla	95.6 ± 24.1	79.50 ± 24.5	−16.8	**0.019**	0.66	2–5	<0.001	0.20
							2–6	0.008	0.94
	(3) Max-Cur	127.8 ± 20.0	95.90 ± 15.5	−24.9	**0.005**	1.78	3–5	<0.001	1.47
							3–6	0.001	0.23
	(4) Max-Pla	129.6 ± 18.5	107.63 ± 22.8	−16.9	**0.025**	1.06	4–5	<0.001	1.58
							4–6	0.007	0.31
	(5) C-Pla	90.5 ± 29.1	101.25 ± 33.3	11.9	**0.036**	0.36			
		
	(6) C-Cur	121.9 ± 30.6	119.20 ± 28.0	−1.9	0.638	0.09			
		
Total Cholesterol (mg/dL)	(1) Aecc-Cur	188.4 ± 31.3	169.91 ± 35.1	−9.8	**0.037**	0.56	1–5	<0.001	0.34
							1–6	0.003	0.77
	(2) Aecc-Pla	195.3 ± 35.9	178.93 ± 34.5	−8.4	**<0.001**	0.46	2–5	<0.001	0.29
							2–6	0.038	0.90
	(3) Max-Cur	219.9 ± 29.7	191.70 ± 36.9	−12.8	**0.005**	0.84	3–5	<0.001	1.55
							3–6	<0.001	0.26
	(4) Max-Pla	199.8 ± 34.1	181.30 ± 33.2	−9.3	**0.005**	0.55	4–5	<0.001	1.64
							4–6	0.006	0.39
	(5) C-Pla	208.8 ± 31.4	215.75 ± 29.7	3.3	**0.003**	0.24			
		
	(6) C-Cur	177.2 ± 32.5	173.93 ± 30.7	−1.8	0.604	0.11			
		
High Density Lipoprotein (mg/dL)	(1) Aecc-Cur	60.1 ± 13.9	67.2 ± 12.0	11.9	**<0.001**	0.54	1–5	<0.001	0.58
							1–6	0.001	0.47
	(2) Aecc-Pla	51.3 ± 11.6	57.1 ± 10.5	10.6	**0.009**	0.52	2–5	0.007	0.26
							2–6	0.014	0.16
	(3) Max-Cur	57.3 ± 7.7	67.9 ± 12.1	18.5	**0.011**	1.04	3–5	<0.001	0.44
							3–6	<0.001	0.34
	(4) Max-Pla	61.0 ± 14.6	70.7 ± 15.1	15.9	**0.005**	0.68	4–5	<0.001	0.68
							4–6	<0.001	0.57
	(5) C-Pla	65.9 ± 11.2	62.5 ± 10.8	−5.1	0.146	0.31			
		
	(6) C-Cur	53.1 ± 11.0	54.5 ± 9.7	2.6	0.063	0.16			
		
Low Density Lipoprotein (mg/dL)	(1) Aecc-Cur	116.9 ± 27.1	105.0 ± 25.7	−10.2	**<0.001**	0.45	1–5	<0.001	0.45
							1–6	0.004	0.36
	(2) Aecc-Pla	111.5 ± 16.9	102.3 ± 26.4	−7.4	**0.008**	0.33	2–5	<0.001	0.28
							2–6	0.015	0.19
	(3) Max-Cur	142.9 ± 11.6	121.9 ± 16.5	−13.6	**0.012**	0.63	3–5	<0.001	0.63
							3–6	0.003	0.53
	(4) Max-Pla	134.5 ± 26.7	116.1 ± 28.1	−13.7	**0.005**	0.65	4–5	<0.001	0.56
							4–6	0.004	0.46
	(5) C-Pla	125.6 ± 25.0	130.6 ± 25.1	4.0	**0.016**	0.21	5–6	0.047	0.13
		
	(6) C-Cur	108.6 ± 17.5	106.8 ± 15.8	−1.7	0.057	0.12			
		

Data are presented as means ± standard deviations. Significant pairwise time differences are highlighted in bold. Δ% represents the percent change between pre- and post-intervention, calculated as [(post-test score − pre-test score)/pre-test score] × 100. ES, Hedge’s g effect size. Aecc-Cur, accentuated eccentric training + curcumin supplementation group; Aecc-Pla, accentuated eccentric training + placebo group; Max-Cur, maximum strength training + curcumin supplementation group; Max-Pla, maximum strength training + placebo group; C-Pla, passive control + placebo group; C-Cur, passive control + curcumin supplementation group.

**Table 5:** Clinical relevance of the study for the intervention groups.

Variable	End-Point/Cut-Point	Reference	Benefits/Risks	Aecc-Cur (*n* = 16)	Aecc-Pla (*n* = 13)	Max-Cur (*n* = 10)	Max-Pla (*n* = 10)	C-Cur (*n* = 15)	C-Pla (*n* = 17)
Fat mass	5% decrease	Poobalan et al., 2004 [68]	A 5% weight loss significantly improves obesity-related cardiovascular and metabolic health	87%	69%	100%	100%	0%	0%
Muscle mass	1% increase	Oh et al., 2021 [69]	Decrease of 19% in the metabolic syndrome	100%	100%	100%	80%	7%	0%
Systolic blood pressure	10 mm/Hg decrease	Law et al., 2009 [70]	Decrease of 21% in cardiovascular disease risk and 46% in stroke	81%	92%	90%	70%	7%	0%
Diastolic blood pressure	5 mm/Hg decrease	Law et al., 2009 [70]	Decrease of 21% in cardiovascular disease risk and 46% in stroke	75%	54%	100%	90%	7%	0%
Glycemia	7.92 mg/dL or 0.44 mmol/L decrease	Keutmann et al., 2020 [71], Qiao et al., 2021 [72]	Decrease of 14% in stroke	63%	69%	70%	50%	7%	0%
Creatinine	0.97 mg/dL cut-point ♀ 1.15 mg/dL cut-point ♂	Odden et al., 2009 [73]	≤0.97 ♀ or ≤1.15 ♂ decrease odds of functional limitation	75%	77%	80%	60%	79%	87%
Triglycerides	40 mg/dL	Marston et al., 2019 [74]	Decrease of 5% in cardiovascular disease risk	6%	0%	30%	20%	0%	0%
Total cholesterol	23.20 mg/dL or 0.60 mmol/L decrease	Mann et al., 2014 [75]	Decrease of 19% in the cardiovascular disease	31%	30%	60%	20%	0%	0%
High-Density Lipoprotein	1 mg/dL or 0.03 mmol/L increase	Gordon et al., 1989 [76]	Decrease of 3% in the cardiovascular disease risk and 4.7% in cardiovascular disease mortality	100%	77%	90%	100%	60%	18%
Low-Density Lipoprotein	38.66 mg/dL or 1.00 mmol/L decrease	Ference et al., 2012 [77]	Decrease of 20–25% in cardiovascular disease risk	17%	0%	0%	0%	0%	0%

Aecc-Cur, accentuated eccentric training + curcumin supplementation group; Aecc-Pla, accentuated eccentric training + placebo group; Max-Cur, maximum strength training + curcumin supplementation group; Max-Pla, maximum strength training + placebo group; C-Cur, control + curcumin supplementation group; C-Pla, control + placebo group; ♀, women; ♂, men.

## Data Availability

Data used in this study are part of a larger ongoing project. Access to the data can be provided upon reasonable request to the corresponding author.

## Abbreviations

The following abbreviations are used in this manuscript:

1RM	One-repetition maximum
Aecc	Accentuated eccentric
ANCOVA	Analysis of covariance
ANOVA	Analysis of variance
BMI	Body mass index
C	Control group
Cur	Curcumin
ES	Effect sizes
HDL	High-density lipoprotein
LDL	Low-density lipoprotein
Max	Maximal strength group
MCID	Minimal clinically important difference
MetS	Metabolic syndrome
MET	Metabolic equivalent of task
Pla	Placebo group
RPE	Rating of Perceived Exertion
RT	Resistance training
